# Hospital and Institutional News

**Published:** 1917-05-26

**Authors:** 


### HOSPITAL AND INSTITUTIONAL NEWS.

### A NATIONAL SYSTEM OF MILITARY HOSPITALS.

SIR JAMES KINGSTON FOWLER has in two letters to the *Times* outlined a scheme for the creation of a truly national system of hospitals for home and foreign service. He takes as the starting-point the medical school, and he suggests that at each school should be formed a military hospital named after the school. It should be called "St. Mary's No. 1 Hospital" or "Cambridge No. 1 Hospital," according to its place of origin. The senior members of the staff would supply the visiting staff of the hospital. This No. 1 hospital would be intended entirely for home service, but a No. 2 hospital would be created for foreign service, staffed by the junior members of the staff of the "mother hospital," and in the same way a No. 3 hospital would be formed if necessary. The nursing staff in each case would be derived so far as possible from those nurses who had been trained in the hospital whence these units are derived. Sir James does not appear to suggest that this organisation should be perfected during the present war, but that it should be worked out when peace comes so as to be available when it shall be needed. He urges that it would foster the *esprit de corps*, and so would raise the standard of work both in the medical and nursing staffs. There is much to be said for the idea, but it is almost impracticable to do anything along these lines until the war comes to an end.

### THE RED CROSS SERVICE IN THE ABBEY.

THE Service of Thanksgiving at Westminster Abbey on May 17, in which deserved honour was paid to the British Red Cross Society and the Order of St. John of Jerusalem, had been arranged with evident care and much thought by the Dean of Westminster, Bishop Ryle, and it included special features which Westminster Abbey seldom admits. The two most noticeable and rare features of the service were the procession, preceded by Sir Frederick Bridge and the trumpeters, led by the Cross-bearer, who with the choir and clergy passed under the choir screen, turned northward, and made a complete circuit of the ambulatory, to re-enter the choir under the screen, the hymn sung being "For all the Saints," to music which was not familiar to the majority of those present, though it was well chosen, and the effect was grateful and inspiriting. The *Times* affirms that within the Abbey it was hard to say whether eye or ear received the nobler impression. The scarlet cassocks of the Abbey choir and the robes of the clergy glowed against a background of ancient grey stone. As the choir drew near or went further off, passed out of sight behind the high altar or flowed full up the centre of the choir, the effect of the music, voices, organ, and trumpets was august and beautiful. Both the choir and clergy were finally massed in the sacrarium, east of the altar rails, and high above the congregation, where the anthem was sung, which Sir Frederick Bridge has composed on the words which Shakespeare puts into the mouth of King Henry VI.:—

> God's goodness hath been great to thee.
> Let never day nor night unhallowed pass,
> But still remember what the Lord hath done.

The Dean said the prayer for Victory, and the service closed with the National Anthem, or rather with one verse only, and the general feeling was one of disappointment or something more, that the words of the whole Anthem should not have been sung, for what occasion could have been more appropriate? Why, indeed, is it that our churches and others are getting into the habit of treating the National Anthem with disrespect by omitting the greater part of it? We are moved on behalf of people of all classes to protest against the course taken at the Abbey. We venture to express the hope that those in authority will mend their ways, and not, for any reason, lend themselves to a course which the people properly regard as a want of patriotism out of sympathy with the spirit of British men and women of all classes in existing circumstances. We hope the general sense of the congregation was sufficiently strong to make itself felt by the Abbey authorities, and that such an omission may never be permitted again in the history of Westminster Abbey.

### MANCHESTER ROYAL INFIRMARY.

INCLUDED in Their Majesties' Lancashire tour was a visit by the Queen to the Manchester Royal Infirmary and its soldier patients, of whom there are upwards of 500 at the present time, the total admitted since the outbreak of the war having been 5,750. Immediately upon the arrival of Her Majesty the following officials were presented: The chairmen of the board of management, the house and finance committees, and of the medical board; the senior physician, the senior surgeon, and the senior surgeon of the special departments; the lady superintendent of nurses, and the general superintendent. The Queen then visited five of the wards occupied by soldiers, the corridors throughout being lined by convalescent soldier patients, residents, students, nurses, and the clerical and domestic staffs, both male and female. By purchase, loan, and gift the wards had been made to look their brightest with spring flowers, whilst Her Majesty's kind words of solicitude addressed personally to many of the sufferers made her visit very memorable.

### HOSPITAL SITE CONVERTED INTO TRENCHES.

ON the site, in the very heart of Manchester, where the old Royal Infirmary stood, patients from the military hospitals at Heaton Park have constructed a reproduction of front-line trenches, complete with parapet, parados, traverses, sandbags, gun-emplacements, bomb stores, dug-outs, signal stations, communication trenches, first-aid posts, and everything necessary to show the citizens of Manchester what life at the Front is like. So, for the time being, the infirmary site is being utilised in a way which can offend none of those with pet projects to advance for the ultimate destination of this vacant plot of land, bought by the Corporation, when the old Royal Infirmary was pulled down, for £400,000. By charging an admission fee to see the "Piccadilly Trenches" it is hoped to raise not less than £10,000 for the Red Cross. It had been hoped to have the show ready for inspection by the King and Queen on their recent tour, but either the sticky soil of Manchester was harder to work in than had been thought, or the muscles of the invalid soldiers were too soft, and the opening has had to be deferred until Whit week.

### THE QUEEN'S HOSPITAL FOR CHILDREN AND THE LORD MAYOR.

THE Mansion House meeting last week, when the work of the Queen's Hospital for Children, in Hackney Road, was considered at the annual meeting, is the second in successive years which has been held there. In May last year, when Colonel Sir C. C. Wakefield was Lord Mayor, his appeal on the hospital's behalf raised £2,500 for its benefit. This year the present Lord Mayor, Sir W. H. Dunn, presided at the annual meeting in the same historic surroundings, and said that he knew something of hospital statistics, and in the light of that knowledge congratulated the committee on keeping down the expenses as much as they had. He hoped that his successors would permit the Queen's Hospital the use of the Mansion House to appeal in support of their work. If they do, and the Queen's has established a courtesy claim to it, an interesting departure will have taken place. The Mansion House has given abundant evidence of its interest in the welfare of voluntary hospitals, which is significant in view of the supposed antagonism between municipalities and the voluntary system; but to allow the annual meeting of a particular hospital always to be held there would be a signal example of generosity. The Queen's Hospital may be congratulated on being the first to gain this privilege.

### THE EMPLOYMENT OF ALIEN DOCTORS AT HOSPITALS.

SENTIMENTAL objections to the employment of coloured medical men as hospital house surgeons and in other capacities in the health service of this country are among the many things which the war has swept away, and the new ideas which have, to a large extent, replaced the old are based upon a recognition of efficiency as the vital factor, race being a thing apart. In the Liverpool district young Indian medical men, educated, of course, in England and with English degrees, have established a reputation in connection with their work at the voluntary hospitals, and of their keen anxiety to serve the Empire at this crisis there is no doubt. At the Liverpool Poor-Law hospitals, too, Indian doctors figure on the attenuated lists of applicants for the vacant house-surgeon posts as they arise. At King Edward VII.'s Hospital, Cardiff, the house surgeon is a Chinese medical man, while the resident surgical officer is an Egyptian. Last week the Newcastle Insurance Committee considered and dismissed protests as largely sentimental against the engagement of a young Egyptian doctor to attend 3,000 panel patients in the Benwell district of the city, in the absence on war service of the resident medical practitioner. Foreign doctors, too, have in not a few instances filled vacancies arising in consequence of the war. There is on the staff at the Welsh Metropolitan War Hospital at Whitchurch, near Cardiff, a Portuguese surgeon, who was delighted to be able to engage King Manoel in a conversation in his native tongue on His Majesty's recent visit to the institution.

### AN INDIAN ON THE STAFF.

THE only candidate for the vacant post of resident assistant medical officer to the Burnley Union Institution, for which the advertised salary was £300 per annum and the usual allowances, was an Indian. Dr. Mahamarki, the applicant, has been senior officer of the Royal Eye Institution, Manchester, and was interviewed by Dr. Robinson, Mr. Riley, and the Burnley Guardians' clerk. Dr. Mahamarki, however, asked for a salary of £350, and in view of the difficulty in filling the vacancy has gained the appointment. It is noteworthy that the opposition to women doctors, which was fairly widespread before the war, is not the only prejudice that is losing its popularity.

### THE VITALITY OF THE SUNDAY FUNDS.

IT is gratifying to note that on the whole the Hospital Sunday Fund movement throughout the country is maintaining its upward tendency, in spite of this being the third year of the war. It is worth while to remember that this is, strictly speaking, a more remarkable feat than that which has been achieved by the Saturday Fund movement. For the latter was certain to benefit by the creation of the numerous factories, the pressure of war work, the increase in the industrial population of the various cities, and the relatively high wages which rule at present. But the Sunday Fund has none of these important, but perhaps transitory, advantages. It depends, as before, on the churches. It is true that the proceeds of various charity performances, which are added to the church and chapel collections, contribute to this result, and that necessary attention is being paid to secure donations from those who may be absent on the particular Sunday on which a local collection is held. Nevertheless, the vitality of the Sunday Fund movement is shown convincingly, and we have thereby only the latest lesson of the voluntary hospital system—namely, that its strength is measured by the work to be done, not by any supposed limit to public generosity.

### THE OSTEOPATH AGAIN.

THE advocates of weird systems of healing are very much to the fore just now, and the osteopaths, a body of which a good deal was heard some years ago, have joined cause with the bonesetters in demanding the right to exercise their s[illegible]l on our wounded. Apparently, in America there are no fewer than 8,000 holding the qualification of O.D. —or is it D.O.?—and in that happy land public confidence in them is widespread. Their theory (evolved from somebody's inner consciousness and wholly unsupported by scientific proof) is that all human ills are due to some slight displacement of the vertebræ, which exert a corresponding pressure upon the cord beneath. They therefore devote themselves to massage in various intricate forms upon the spinal columns of their patients. The results are said to be marvellous. We must bear in mind, however, that America is the land of universal panaceas. Where Christian Science and Theosophy flourish, there is no accounting for the other nostrums that will obtain universal repute. It is to be hoped that the War Office authorities will not be so influenced by the current talk of professional jealousy among doctors as to allow a band of these faddists loose in our hospitals. The wounded have suffered too much to be made the helpless victims of popular clamour.

### ANOTHER WAR COMPLAINT.

Shell shock, Zeppelin neck, trench fever, and trench feet we know, but we confess that *psychose de fils-de-fer* is new to us. It is apparently the "state of mind produced by prolonged imprisonment behind wire-netting fences," and is one of the maladies that the international commission for the internment of prisoners in Switzerland suggest would be benefited by a change of habitat. This commission, which has been formed as a result of the interchange of views between the Swiss authorities and the French and German Governments, has put forward a number of proposals which, it is hoped, will facilitate the repatriation of prisoners of war. Men who are above a certain age, have been more than eighteen months in captivity, or are so disabled that they will not be able to return to the lines for more than twelve months, if at all, are to be repatriated; the claims of each case are to be adjudged by a committee composed of three officers of the Swiss Medical Corps and three from the French or German service. Those who are likely to recover within twelve months are to be confined in Switzerland, a change likely to promote their speedy recovery. With all this we entirely agree. But proposal five is so extraordinary that we can only presume it to have been mistranslated. "To exchange prisoners automatically, perhaps once a month, on the understanding that all the men so exchanged after April 17 this year shall not be employed either at or behind the Front." With a chivalrous enemy this counsel of perfection might conceivably be followed, but to expect a foe such as the Germans to adhere to it would be to prolong the war to the point of mutual annihilation.

### A REVISED TRUST DEED FOR BLACKPOOL HOSPITAL.

After many months of discussion, Mr. W. Ross, who presided at the annual meeting of the Blackpool Victoria Hospital, was able last week to propose the approval of the revised trust deed. The old abuses and the impasse to which they led were fully described in The Hospital at the time. Mr. Ross remarked that a sub-committee of the board and a sub-committee of the Corporation had arrived at a revision which he described as reasonable. The Corporation, in fact, had given up the voting power which as donors it had previously held. Under the proposed revision the number of the board would be increased to eighteen, to which the medical staff would appoint four and the Corporation three. The revised deed, the adoption of which was seconded by Mr. Read, was declared by Dr. Brown to be inadequate, since the revision, he said, had not gone far enough for him. The alterations, however, were approved, and the retiring members of the board were re-elected. The Corporation seems to have shown not discretion only but generosity, since, with the support of the Mayor, it has given an additional grant of £250 to the hospital during the past year. A resident woman house surgeon, it is hoped, will be appointed shortly.

### THE REWARD OF A COURAGEOUS APPEAL.

We are glad to be officially informed of the remarkable success which has attended the courageous appeal for £10,000 lately issued on behalf of the Royal Southern Hospital, Liverpool. As we remarked at the time, the subject of the appeal was the dullest possible, the payment of a large debt due to the bank. Nevertheless, in spite of an appeal for £20,000 on behalf of the local branch of the British Red Cross Society, of another on behalf of the French Wounded Emergency Fund, and of two more, both connected with war charities, and all promulgated before the Royal Southern entered the lists, the sum required was raised with gratifying promptitude. In fact, the total sum subscribed hardly fell short of £11,000. Mr. John S. Birbeck and his board may well be proud of this result, which is a tribute to their good management, since success is rather a science than a gamble, and requires hard work and ability to ensure its result. Mr. Birbeck, who is now acting superintendent, deserves congratulation both for the success and the example which it sets to his *confrères* in other institutions at the present time.

### WORKHOUSE MASTER AS OFFICER IN CHARGE OF A MILITARY HOSPITAL.

In spite of the protest of Mr. A. F. Sutcliffe, who declared the Primrose Bank Military Hospital to be as well conducted as any other in the country, the Burnley Board of Guardians has passed a resolution requesting the military authority to appoint a commissioned officer to take charge of the hospital in order that better discipline may be maintained. It appears that the master of the workhouse has been acting as officer in charge, and the Guardians point out that, if he were relieved of those duties, he would be able to give more time to his Poor-Law duties. Mr. Sutcliffe suggested that the reason for this resolution was merely that the men had been somewhat lax in returning to hospital at the regulation hour of 8 p.m. But what are we to say of the principle which places the discipline of a military hospital in the hands of a workhouse master? What about wounded soldiers and the Poor Law? What about hospital administration? In civil life the best proved of all rules is that which insists that the separation of the infirmary from the workhouse shall be absolute.

### HOSPITALS IN MUNITION AREAS.

People are beginning to realise that the length of the war is a factor to be weighed when considering the possibility of issuing appeals. Some of them cannot be postponed indefinitely, for the need is too great, and if building cannot be at present undertaken building funds can and should be collected when the need is one which must be met by bricks and mortar as soon as peace returns. It is doubtless such considerations as these which have led to the issue of an appeal for £35,000 on behalf of the Miller General Hospital for South-East London. It seems a large sum, but £4,500 or more represents a debt on the new wards, and a sum of £20,000 is required for the rebuilding of the nurses' home and the out-patient department, which at present are accommodated in some old houses. In addition to this the annual expenditure amounts to £10,000, of which less than one-tenth is provided out of the income from invested funds. Since the hospital serves Woolwich, Greenwich, and Lewisham, and is thus in the heart of one of the munition-making districts, its appeal cannot be neglected, and we hope that once again will be proved by its success that the voluntary hospitals' work is adequately recognised by the generous public.

### NEW AUXILIARY HOSPITALS IN WALES.

The demand for increased accommodation has led to the opening in South Wales recently of a number of new auxiliary hospitals. Among these are the following: Glanrhyd, Pontardawe, containing 90 beds, which has been placed at the disposal of the St. John Association by the relatives of the late Mr. Arthur Gilbertson; Stebonheath, Llanelly, 175 beds; St. Michael's College, Llandaff, 175 beds; The Beeches, Caerphilly, 80 beds, lent by Mr. Pigott; Pontardawe Workhouse Infirmary, 60 beds; and the Quarr, Clydach, 40 beds, lent by Mrs. Percy Player. Patients are already in occupation of most of these new hospitals.

### THE ROYAL GWENT HOSPITAL, NEWPORT.

Proposing the adoption of the report at the annual meeting of the directors of the Royal Gwent Hospital, Newport, last week, Sir Garrod Thomas, the chairman, made a mild protest against the inadequate sum paid by the authorities for the treatment of sailors and soldiers, viz., 2s. a day, which, he pointed out, is insufficient to meet the expenses. The hospital's income increased from £11,920 in the previous year to £12,070; but its burdens increased also, for the expenditure jumped from £10,775 to £13,286; and now it is faced with an accumulated deficit of £3,741. Reference was made to the loyalty and self-sacrifice of those "left behind" for what they have done while the other doctors are at the Front, and Sir Garrod Thomas, in a tribute to the work of medical men in the war, observed that their efforts had been more successful than in any previous conflict. Lord Tredegar was re-elected president of the Royal Gwent Hospital.

### HAMPSTEAD GENERAL HOSPITAL SECRETARYSHIP.

The secretaryship of the Hampstead General Hospital, vacated by Mr. Byrne, has been filled by the appointment of Mr. Harold Wigg, secretary of the Empire Hospital for Paying Patients, Ltd., Vincent Square, Westminster, now a special hospital for military officers. Mr. Wigg's association with hospital work extends over a period of twenty-three years, his early experience being acquired under the secretary of the Croydon General Hospital, Mr. J. Jones, in whose service he became associated with other institutions, including the Whitgift Foundation. For six years Mr. Wigg was secretary of the Walsall and District Hospital (Sister Dora's Hospital), returning to London five years ago, and he subsequently became secretary to the Empire Hospital. We look forward with interest to Mr. Wigg's work at Hampstead, and wish him every success and the best of health to develop its many interests to the full.

### HOSPITAL SECRETARY FOR THE ARMY.

At the Richmond Tribunal recently Mr. Richard Allen, the secretary of the Richmond Royal Hospital, appeared on an adjourned review of his certificate of exemption. The chairman of the hospital committee, Mr. Landover, said the committee had decided not to oppose the application of the military representative; he left it to the Tribunal whether Mr. Allen would be better as secretary of the hospital or on military service. He also said they would be able to make arrangements for carrying on the work while Mr. Allen is away, but asked that the calling up might be postponed until July 1. This very moderate request was agreed to by the military representative, and the Tribunal thereupon withdrew the certificate of conditional exemption. Mr. Landover added that Mr. Allen, who is thirty-nine years of age, has been placed in Class A; and that he is a most efficient secretary whose services will be greatly missed.

### MALE V.A.D.s AND MILITARY SERVICE.

Some degree of astonishment seems to have been created in Glasgow by the recent refusal of a Military Appeal Tribunal to exempt from military service a man of military age who is the commandant of a Voluntary Aid Detachment of the British Red Cross Society. The normal occupation of this individual is stated to be that of an attendance officer under the Glasgow School Board; and in his spare time he carries out the duties of commandant of a V.A.D. detachment. The Red Cross Society and the V.A.D. detachment seem both to have appealed on his behalf, but were very properly told that as they are not the man's employers they had no *locus standi* before the Tribunal. Even a request for postponement was refused, and very properly too. It is difficult to understand why the Red Cross Society or its local branch should go out of its way to appeal on behalf of a case such as this; on the face of the matter, as reported in the local press, the odd thing is that the man was not taken for service long ago. An idea has evidently sprung up that V.A.D. commandants of military age are immune from service; the decision in this instance should go far to explode this erroneous notion.

### DISABLED FIGHTERS' WORKSHOPS.

We are glad to learn that the Lord Roberts Memorial Workshops, which have been established to enable disabled soldiers to support themselves and their families independently of pension grants, are now in a fair way to becoming a paying concern. At the moment 840 men are employed, but the committee are making active preparations to deal with 5,000. A notable part of these training-shops is the beneficial effect upon the men themselves of having some active employment instead of remaining idle and brooding over their misfortunes. In some cases the earning capacity of the men is actually higher than it was before their disablement. For example, a farmer's boy who before the war was earning 15s. a week, lost his right arm. He now works at the circular saw and is in receipt of 29s. weekly. Those who have lost both arms are engaged as guides about the workshops, and make 25s. a week. In the doll factory a serious effort is being made to establish the supremacy of the British doll over the German article. There seems little doubt that the dolls turned out by these crippled men can compare favourably with the finest that ever came out of Germany. It is hoped later to establish these workshops all over the country, so that those who wish to work near their homes can do so.

### THE DAVID LITTLE HOMESTEAD MEMORIAL.

The scheme of the Homestead Association for helping disabled officers and men of the Navy and Army has received an important gift with the presentation by Mrs. Little, in memory of her late husband, Dr. David Little, the Manchester oculist, of the Bank House Estate at Congleton. Only a dozen miles or so from Buxton, the estate, which comprises some twenty acres, includes a roomy house, well-timbered grounds, and a farm with house and buildings. This gift, with its pleasant association with the name of the late Dr. Little, will help the Association in its objects of providing disabled soldiers with home comforts and such training, especially in agriculture, as they may be able to undergo. The Association also hopes to co-operate with other societies in finding employment for those whose disablement would ordinarily stand in their light. In this way it is hoped that men will not be obliged to spend their pensions until they are completely recovered, when the sum thus accumulated will provide them with a small capital at their start once more in the world. The house will be known as the David Little Homestead Memorial.

### A MEMORIAL TO MISS EMILY JACKSON.

The life-work of Miss Emily Jackson, the founder, administrator, and maintainer of the Children's Hospital for Hip Disease at Sevenoaks, was described in these columns at the time of her decease full of years and honour (The Hospital, December 30, 1916, p. 257). It is only fitting that the memory of this wonderful woman should be perpetuated by some specific effort of others: her name and record have been made imperishable by herself, of course, but the community at large owes her some further definite recognition of her labours. This is to be accomplished, we are pleased to learn, if a proposal be carried through whereby a plot of land adjoining the hospital is to be purchased and the grounds already attached to the institution are to be levelled. The appropriateness of extending the sphere of usefulness of her own hospital by adding to the amenities which surround it is undeniable; and it can scarcely be doubted that the response to the appeal for this scheme will be immediate and satisfactory.

### A "CHILDREN'S BED" AT PORTH COTTAGE HOSPITAL.

Sir William James Thomas once more showed his generous interest in hospitals at a gathering at Treherbert the other day, when he and Lady Thomas were the recipients of a wedding gift from the officials and workmen of the Ynysfaio Colliery. At the suggestion of his wife, and in order to mark her appreciation of the part local children had taken in the demonstration, Sir William announced his intention of contributing 1,000 guineas to the Porth Cottage Hospital, to endow a bed to be called "The Treherbert Children's Bed." Sir William also mentioned that the new ward of fourteen beds at the Porth Hospital would soon be completed, and would then be handed over to the committee. In acknowledging a cheque for £70 raised by Ynyswen school children, by means of a concert, for the endowment for one year of a Treherbert bed at the Welsh Hospital, Netley, Sir W. J. Thomas said he would himself endow a second bed in the name of the Ynyswen children. Up to the beginning of May 4,987 wounded soldiers had been treated at Netley Welsh Hospital, and, as treasurer of the hospital, he was proud to be able to say that Rhondda residents maintained forty-five of the beds.

### RED CROSS EXPANSION IN DEVON.

In response to the call of the authorities, a very large extension of hospital accommodation has recently been made in Devonshire. The Red Cross Society has added to its total something like 1,000 additional beds, so that the county, if occasion arises, is in a position to accept in all about three thousand wounded men. A new hospital is being set up at Plymouth, and smaller ones opened, or existing ones extended, in various other centres. One of the latest of the V.A.D. institutions is Follaton House, Totnes, a handsome residence, accommodating fifty beds, which has been placed at the disposal of the Red Cross Society by Mr. and Mrs. Stanley Carey. Apart from considerable gifts of furniture and equipment from local sources, from the Linen League at Exeter, and the Stores Department of the Red Cross Society, the expense of preparing Follaton House for its new purpose has been about £600, £200 of which was subscribed locally. The institution is being worked as part of the older hospital—Strathmore (twenty-two beds), lent by Mr. and Mrs. Challoner rent free. Dr. Chapman is the medical officer in charge of both, Miss Hartley is the quartermaster, and Miss Tregaskis has left Strathmore to become matron at Follaton House. The addition of this hospital brings the aggregate number of beds provided in Red Cross hospitals in the Totnes division to 290.

### LIABILITY TO TREAT TUBERCULOSIS.

Apparently the Poor Law is bound to treat cases of consumption. The position seems to be made clear from correspondence between the Newark Guardians and the Nottinghamshire County Council. The Guardians complained that they were frequently compelled to render assistance to insured persons and also to provide institutional treatment, but they considered they ought not to be called upon to provide such treatment for insured persons, for whom the responsibility is on the County Council. The County Council replies that there is no statutory or other responsibility on the County Council to provide treatment in the case of insured or uninsured consumptives. The statute dealing with the matter is an enabling statute only. The Guardians are responsible now, and they have always been responsible, for the provision of institutional or other treatment to destitute persons. No Act of Parliament has yet been passed transferring their obligation to anyone else. To provide for the segregation of advanced cases unsuitable for ordinary sanatorium treatment would involve a very large expenditure, and it is extremely doubtful whether the Government would authorise such an expenditure, at any rate during the next few years.

### SPIRIT DUTY GRANT.

Secretaries of voluntary hospitals have received, or will shortly receive, a circular letter from the Local Government Board instructing them how to proceed when making claims for relief or part relief in respect of the duty paid upon spirits used for medical or surgical purposes. A special form for the return is provided, and the spirits to be detailed thereon should not include methylated spirit or spirit obtained free of duty under Section 3 of the Finance Act, 1902. The limiting phrase "for medical and surgical purposes" is to be regarded as excluding also brandy, whisky, and the like. Applications may now be made in respect of 1915 and 1916, and for the purposes of comparison statistics of the consumption for 1914 must also be given. Figures of spirits used must be allocated as between in-patients and out-patients as accurately as possible. The most important part of the announcement is that a grant will be made only in respect of that part of a hospital's work which is paid for by charitable contributions. Subsidies from State or municipal funds, payments made to the hospital for the supply of nurses, by patients, or by the War Office or Admiralty for the admission and treatment of sailors and soldiers would appear to be the principal items not falling within the category of charitable contributions. Claims must be accompanied by copies of the printed reports of the hospital for three years.

### THE RETENTION OF SUFFICIENT STAFF.

The Metropolitan Asylums Board have been confronted with the serious position of keeping a sufficient staff at their various institutions. Great difficulties have arisen to retain those now in their services, and to fill any vacancies that may arise from time to time. The managers have adopted a scheme of payment of bonuses to those remaining in their employment for six or twelve months, by which it is hoped that they may in the future be free from any of the uncertainties that have faced them in the past. Increases of salaries are also decided upon, with the result that it is estimated that there will be an extra sum of £30,000 to be paid annually to the officers in increased wages and war bonuses. It is well maintained by the managers that the work of their staff is of national importance, and consequently they are applying to the Government for permission to grant their officers badges with the Board's arms and the words "On National Service" on them. Those who have been five years under the Board are to have extended leave during the coming summer vacation, indeed every inducement is to be made to keep the officers in their present positions.

### THIS WEEK'S DRUG MARKET.

Changes in value have, this week as last, been few, but the tendency of prices is still in an upward direction. Phenacetin seems to be still very scarce, and the value is advancing. There is practically no change in the position of quinine. Calumba root is very scarce and dearer. Valerian root is wanted, but very little is offered. Vanillin is dearer. Supplies of sodium salicylate are far from plentiful, and the price is again higher; salicylic acid is also dearer. There is still a good demand for glycerophosphates, but supplies are restricted. On the whole, business has been quieter than usual, this being partly due to the nearness of the Whitsuntide holidays, and partly to the extreme prices ruling for so many drugs.

### TO OUR READERS.

Contributions are specially invited from any of our readers to these columns. They should deal with topical subjects and news. They must be authenticated for the information of the Editor only. The minimum payment if published is 5s. There is no hard-and-fast rule as to space, but notes of about twenty lines in length are preferred.

